# Daily Sitting Time and All-Cause Mortality: A Meta-Analysis

**DOI:** 10.1371/journal.pone.0080000

**Published:** 2013-11-13

**Authors:** Josephine Y. Chau, Anne C. Grunseit, Tien Chey, Emmanuel Stamatakis, Wendy J. Brown, Charles E. Matthews, Adrian E. Bauman, Hidde P. van der Ploeg

**Affiliations:** 1 Prevention Research Collaboration, School of Public Health, University of Sydney, Sydney, Australia; 2 Physical Activity Research Group (UCL-PARG), Division of Population Health, Department of Epidemiology and Public Health, University College London, London, United Kingdom; 3 Centre for Research on Exercise, Physical Activity and Health, School of Human Movement Studies, University of Queensland, Brisbane, Australia; 4 Nutritional Epidemiology Branch, Division of Cancer Epidemiology and Genetics, National Cancer Institute, Bethesda, Maryland, United States of America; 5 Department of Public and Occupational Health, VU University Medical Center, EMGO Institute for Health and Care Research, Amsterdam, The Netherlands; Geisel School of Medicine at Dartmouth College, United States of America

## Abstract

**Objective:**

To quantify the association between daily total sitting and all-cause mortality risk and to examine dose-response relationships with and without adjustment for moderate-to-vigorous physical activity.

**Methods:**

Studies published from 1989 to January 2013 were identified via searches of multiple databases, reference lists of systematic reviews on sitting and health, and from authors’ personal literature databases. We included prospective cohort studies that had total daily sitting time as a quantitative exposure variable, all-cause mortality as the outcome and reported estimates of relative risk, or odds ratios or hazard ratios with 95% confidence intervals. Two authors independently extracted the data and summary estimates of associations were computed using random effects models.

**Results:**

Six studies were included, involving data from 595,086 adults and 29,162 deaths over 3,565,569 person-years of follow-up. Study participants were mainly female, middle-aged or older adults from high-income countries; mean study quality score was 12/15 points. Associations between daily total sitting time and all-cause mortality were not linear. With physical activity adjustment, the spline model of best fit had dose-response HRs of 1.00 (95% CI: 0.98-1.03), 1.02 (95% CI: 0.99-1.05) and 1.05 (95% CI: 1.02-1.08) for every 1-hour increase in sitting time in intervals between 0-3, >3-7 and >7 h/day total sitting, respectively. This model estimated a 34% higher mortality risk for adults sitting 10 h/day, after taking physical activity into account. The overall weighted population attributable fraction for all-cause mortality for total daily sitting time was 5.9%, after adjusting for physical activity.

**Conclusions:**

Higher amounts of daily total sitting time are associated with greater risk of all-cause mortality and moderate-to-vigorous physical activity appears to attenuate the hazardous association. These findings provide a starting point for identifying a threshold on which to base clinical and public health recommendations for overall sitting time, in addition to physical activity guidelines.

## Introduction

The benefits of physical activity for health are well established, with inactivity accounting for 9% of premature mortality globally[1]. Sedentary behavior, as distinct from physical activity, encompasses a broad range of behaviors that involve sitting or lying down and do not increase energy expenditure substantially during waking time[2,3].

Sedentary behavior is very pervasive. On average, adults in Western countries spend between 55% and 70% of their day sedentary, according to objective monitoring[4-6]. This corresponds to approximately 9-11 h/day of sitting. In a multinational surveillance study involving 49,493 adults aged 18-65 years from 20 developed and developing countries, the overall mean reported total sitting time was 5.8 h/day, with quintiles ranging from <3 h/day to ≥9 h/day[7]. A growing body of literature suggests high sitting time is associated with higher risk of adverse health outcomes, including cardiovascular disease, type 2 diabetes, cancer and mortality, even after adjustment for moderate-to-vigorous physical activity[8-13]. A recent large study reported that sitting time accounts for 7% of all deaths in adults aged 45 years and older[10].

In contrast with physical activity, for which there have been clinical and public health guidelines in place for nearly two decades[14], no quantitative guidelines exist for sedentary behavior because it is not known how much sedentary behavior is harmful to health. Recent meta-analytic reviews have begun to explore this issue. Grøntved & Hu [13] conducted a meta-analysis of three studies that included a focus on the associations between TV-viewing and mortality. They found a pooled hazard ratio of 1.13 per 2-hour increase in TV-viewing per day for all-cause mortality. Such evidence has value for formulating quantitative clinical and public health recommendations for TV-viewing time, but not for overall sitting, because TV-viewing time is a poor proxy of total sitting time. In another study, Katzmarzyk and Lee [15] reported an 18%-45% increase in risk of all-cause mortality for higher levels of sitting, relative to the lowest level. One study in this meta-analysis used a qualitative categorical measure of daily sitting (none of the time, 1/4 of the time, ½ the time, three-quarters of the time, all of the time)[16] while the other study measured daily leisure-time sitting[17]. Therefore, the authors could not assess dose-response relationships between total sitting time and mortality. More recently, Wilmot and colleagues [18] reviewed the associations of sedentary time with mortality, and reported an overall 49% increase in all-cause mortality risk for the group with highest sedentary time level compared to the lowest group. However, this meta-analysis of 16 prospective studies involved mixed exposures of sedentary behavior, such that TV-viewing, leisure-time sedentary behavior, and sitting, measured in heterogeneous units and categories, were combined in the same analyses. Once again, the authors were unable to examine dose-response relationships between total sitting time and all-cause mortality.

Hence, the aim of this meta-analysis was to quantitatively summarize the results of all published prospective cohort studies that have examined the association between daily total sitting time and all-cause mortality, and to examine potential dose-response relationships. To the best of our knowledge, this is the first meta-analysis of dose-response relationships between total daily sitting time and mortality risk, with and without adjustment for physical activity.

## Methods

### Information sources and search strategy

This meta-analysis was conducted following the checklist of the Meta-Analyses of Observational Studies in Epidemiology[19]. We used a three-part search strategy to identify potential studies for this meta-analysis: 1) we searched the reference lists of the first systematic reviews that covered the published literature on a range of sedentary behaviors in adults (e.g., sitting, TV-viewing) and multiple health outcomes (e.g., overweight or obesity, cardiovascular disease, diabetes, cancer, mortality) covering a period from 1989 to January 2011 [8,9]; 2) we searched Medline, Pubmed, Embase, and Web of Science for studies published between January 1, 2011 and January 31, 2013 with the following search terms: (("sitting time" OR "sedentary behavior" OR "sedentary behaviour") AND (mortality OR mortalities OR death OR fatal)) AND (risk OR Cox OR hazard OR survival analysis OR odds); and 3) we further searched the reference lists of included papers and later review articles, as well as all authors’ personal literature databases for relevant studies, including publications in press.

### Eligibility criteria

Inclusion criteria were: prospective design; published in English; studies of healthy adult populations at baseline; measured total sitting time as an exposure variable; had all-cause mortality as an outcome variable (i.e., mortality was ascertained without consideration of specific cause of death); provided estimates of relative risk (RR) or odds ratios (OR) or hazard ratios (HR) with 95% confidence intervals (CIs) or reported data for their calculation[13]. Conference abstracts were not included.

### Data extraction

The following data were extracted from retrieved articles: author(s), study name, year of publication, total sitting time measure, sample size, age at baseline, follow-up time, person years, confounding variables that were adjusted for in the analysis, and the HR, RR or OR estimates with corresponding 95% CIs for models with and without adjustment for physical activity. Two authors independently extracted the data from each study and compared the data for consistency. Any discrepancies between the two reviewers were settled through discussion and a third reviewer’s help was sought for resolving disagreements. We contacted corresponding authors to confirm or request missing data and incorporated responses into the analyses.

### Study appraisal

Two authors independently appraised the methodological quality of all included studies using a quality rating list based on previous checklists[20-23]. This rating list consisted of 15 criteria and each criterion was assessed as ‘yes’ (=1) or ‘no’ (=0), with each assigned equal weighting, so that a quality score ranging from 0 to 15 could be calculated for each study. Any disagreements in quality ratings between the two reviewers were resolved in a consensus meeting between them, with a third reviewer consulted in the event of disagreement.

### Analysis

“Dose” was assigned using the midpoint between the lower and upper boundary of each sitting category for which a HR, RR or OR was provided. For open ended categories we assumed the same magnitude of dose as for the neighboring category. For example, the categories <4, 4-<8, 8-<11, ≥11 h/day were assigned doses of 2, 5, 9 and 12 h/day, respectively.

First, for data with and without adjustment for physical activity, we used spaghetti plots to graphically illustrate the shape and direction of the dose-response association between sitting hours and risk of death across all studies[24]. Using the generalized least squares for trend (GLST) procedure we then estimated the trend for each study sample and pooled the estimates to produce a forest plot and random effects trend[25]. A funnel plot was made for visual inspection of publication bias. 

We then used the method of pool-first to estimate the dose-response trend for all study samples combined[25]. Dose-response linear and multiple non-linear models were fitted to determine the model of best fit for the pooled data. Non-linear responses were tested with piece-wise spline regressions using inflection point(s), or “knots”, identified from the model of best fit[26]. Model adequacy was determined by the model chi-square and goodness-of-fit statistics produced by the GLST procedure. 

Statistical heterogeneity was tested by calculating the I^2^ statistic and its interpretation was based on the Cochrane Collaboration interpretation whereby 50% or greater represents substantial heterogeneity[27].

We calculated the population attributable fraction (PAF) for each included study following methods outlined by Katzmarzyk and Lee[15]. An overall weighted PAF estimate was computed based on the weights from the meta-analysis. All analyses were carried out with STATA version 11 (STATA Corporation, College Station, TX, USA).

For the purposes of this paper, we refer to daily sedentary time and sitting time as ‘daily total sitting time’, and moderate-to-vigorous physical activity as ‘physical activity’, unless explicitly stated otherwise.

## Results

### Study selection

**Figure 1:**
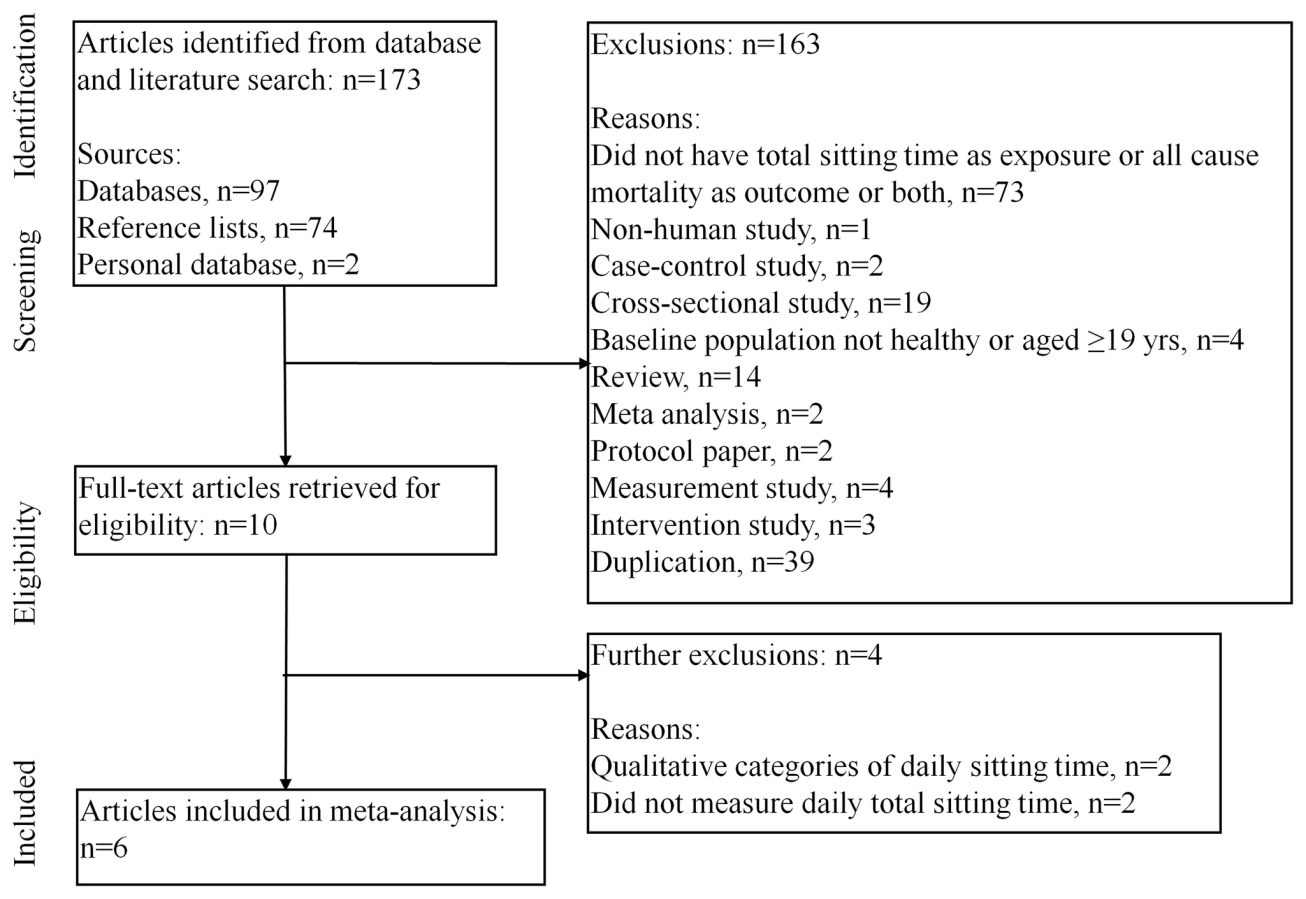
Selection of studies for inclusion in the meta-analysis.

The literature search yielded a total of 173 abstracts (Figure 1). One hundred and sixty three did not meet the core inclusion criteria and were excluded initially, and ten full-text articles were further considered. Four studies were subsequently excluded because they did not measure daily total sitting time[16,17,28,29]. Only one study, by Koster et al, objectively assessed total sedentary time using accelerometers[30]. As accelerometers have been shown to measure sitting time in a different manner to questionnaires[31], we ran models with this study included and excluded, to see if the results were changed with inclusion of this paper. Two papers were included from authors’ personal literature databases[32,33]. Therefore, a total of six studies was included in the meta-analysis. 

### Study characteristics

The characteristics and main outcomes of the prospective cohort studies included in this meta-analysis are shown in Table 1. 

**Table 1:** Characteristics and results of the studies included in the meta-analysis.

**Study**	**Total sitting measure**	**Sample**	**Follow up mean (SD)**	**Outcomes: All-cause mortality**	**HR (95%CI)**	**p or HR for trend**	**N**	**No deaths**	**Person-years**	**Adjustments**
Japan Public Health Center Study (JPHC) (1995-1999), Inoue et al 2008 [34]	Daily sitting (h/day categories); Average time spent per day in sedentary activity (<3, 3-<8, 8+ h/day)	83,034 adults aged 45-74 yrs, 52.8% female	8.7 yrs	PA ADJUSTED						Age, geographic area, occupation, history of diabetes, smoking, alcohol intake, BMI, total energy intake, heavy physical work or strenuous exercise (none, <1 h, ≥1h), walking or standing hours (<1 h, 1–3 h, ≥3 h), and leisure-time sports or physical exercise (<1 day/week, 1–2 days/week, ≥3–4 days/ week)
				Men						
				<3	1.00	p=0.036	17667	1331	152673	
				3-8	1.02 (0.95-1.11)		18223	1445	156183	
				>=8	1.18 (1.04–1.35)		3293	322	28491	
				Women						
				<3	1.00	p=0.698	19651	648	173068	
				3-8	0.95 (0.85-1.06)		21404	704	189268	
				>=8	1.10 (0.82-1.25)		2796	114	25389	
				NOT PA ADJUSTED	Not reported	Not reported	Not reported	Not reported	Not reported	Not reported
NIH-AARP Diet and Health Study (1995-1996), Matthews et al 2012 [11]	Overall sitting (h/day categories); “During a typical 24-hour period over the past 12 months, how much time did you spend sitting?” (<3, 3-4, 5-6, 7-8, 9+ h/day)	240,819 adults aged 50-71yrs, 41-48% female across sitting categories	8.5 yrs (1.7)	PA ADJUSTED						Age, sex, race, education, smoking history, diet quality, leisure-time moderate-to-vigorous physical activity (never/rarely; <1, 1–3, 4–7, >7 h/wk)
				<3	1.00	p<0.001	48567	3310	415795.71	
				3-4	0.98 (0.95–1.03)		70039	5029	598016.25	
				5-6	1.03 (0.98–1.08)		66705	4851	567142.49	
				7-8	1.02 (0.96–1.07)		35420	2362	300877.13	
				>=9	1.19 (1.12–1.27)		20088	1492	169699.7	
				NOT PA ADJUSTED						Age, sex, race, education, smoking history, diet quality
				<3	1.00	p<0.001	48567	3310	415795.71	
				3-4	1.00 (0.96-1.04)		70039	5029	598016.25	
				5-6	1.06 (1.01-1.10)		66705	4851	567142.49	
				7-8	1.06 (1.01-1.12)		35420	2362	300877.13	
				>=9	1.30 (1.22-1.38)		20088	1492	169699.7	
45 & Up Study (2006), van der Ploeg et al 2012 [10]	Total sitting (h/day); “About how many hours in each 24-hour day do you usually spend sitting?”; Categorised later into <4, 4-<8, 8-<11, 11+ h/day	222,497 adults aged at least 45 yrs, 52.4% female	2.8 yrs (0.9)	PA ADJUSTED						Age, sex, education, marital status, urban/rural residence, walking and other moderate-to-vigorous physical activity (0, 1-149, 150-299, ≥300 min/wk), BMI, smoking status, self-rated health and receiving help with daily tasks for long term illness or disability
				<4	1.00	HR= 1.11	58534	1125	164795	
				4-8	1.02 (0.95–1.09)	(95%CI:	107994	2489	302552	
				8-<11	1.15 (1.06–1.25)	1.08-1.15)	41646	1142	114961	
				>=11	1.40 (1.27–1.55)		14323	649	39386	
				NOT PA ADJUSTED						Age, sex, education, marital status, urban/rural residence, BMI, smoking status, self-rated health and receiving help with daily tasks for long term illness or disability
				<4	1.00	HR= 1.14	58534	1125	164795	
				4-8	1.02 (0.95-1.10)	(95%CI:	107994	2489	302552	
				8-<11	1.20 (1.10-1.30)	1.11-1.18)	41646	1142	114961	
				>=11	1.51 (1.37-1.70)		14323	649	39386	
NHANES (2003-04), Koster et al 2012 [30]	Accelerometer-measured sedentary time during waking hours; Actigraph uniaxial accelerometer (AM-7164) worn for 7 consecutive days; non-wear time defined as intervals of ≥60 consecutive minutes of 0 counts with allowance for up to 2 min of counts between 1-100; Sedentary time defined as <100 count/min during wear time	1906 adults aged at least 50 yrs, mean age 63.8, 54% women	2.8yrs	PA ADJUSTED Quartiles of sedentary time (h/day), (1 lowest, 4 highest)		-				Adjusted for gender, age, race/ethnicity, educational level, smoking status, alcohol intake, BMI, diabetes, coronary heart disease, congestive heart failure, cancer, stroke, mobility limitation, and moderate-to-vigorous physical activity (min/day)
				1	1.00 (ref)		476	11	1311.38	
				2 [M:7.6; W:7.2]	1.74 (0.81-3.73)		477	24	1335.998	
				3 [M:9.2; W:8.7]	2.74 (1.35-5.52)		477	41	1283.925	
				4 [M:10.8;W:10.1]	3.26 (1.59,6.69)		476	69	1305.033	
				NOT PA ADJUSTED		-				Adjusted for gender, age, race/ethnicity, educational level, smoking status, alcohol intake, BMI, diabetes, coronary heart disease, congestive heart failure, cancer, stroke, mobility limitation
				1	1.00 (ref)		476	11	1311.38	
				2 [M:7.6; W:7.2]	1.98 (0.95-4.13)		477	24	1335.998	
				3 [M:9.2; W:8.7]	3.31 (1.56-7.03)		477	41	1283.925	
				4 [M:10.8;W:10.1]	4.13 (1.89-9.05)		476	69	1305.033	
Australian Longitudinal Study on Women’s Health (2002), Pavey et al 2012 [32]	Self-reported total sitting time (h/day); “Think about all the time you spend sitting EACH DAY while at home, at work, while getting from place to place or during your spare time. How many hours EACH DAY do you typically spend sitting down while doing things like visiting friends, driving, reading, watching television, or working at a desk or computer on (a) a usual week-day and (b) a usual weekend-day”	6656 women aged 76-81yrs, N=4753 all participants with complete data for all covariates	Median 6yrs (72.3 months)	PA ADJUSTED All P’s with complete data for all covariates (model 5)						Age, education, marital status, area of residence, smoking, alcohol consumption, BMI, walking and moderate-to-vigorous physical activity (<450, ≥450 METxmin/week)
				0-4	1.00	HR=1.05	1286	351	6727.1	
				4-<8	0.97 (0.85-1.10)	(95% CI:	2671	698	14108.9	
				8-<11	1.42 (1.19-1.69)	1.04-1.07)	595	213	2956.7	
				>=11	1.70 (1.40-2.15)		201	102	927.0	
				NOT PA ADJUSTED (model 4)						Age, education, marital status, area of residence, smoking, alcohol consumption, BMI
				0-4	1.00	HR= 1.06	1286	351	6727.1	
				4-<8	0.97 (0.85-1.11)	(95% CI:	2671	698	14108.9	
				8-<11	1.47 (1.24-1.74)	1.04-1.08)	595	213	2956.7	
				>=11	1.73 (1.38-2.18)		201	102	927.0	
HUNT3 Study (2008), Chau et al 2013 [33]	Total sitting (h/day); “About how many hours do you sit during an average day? (include work hours and leisure time)”; Categorised later into <4, 4-<7, 7-<10, 10+ h/day	42,077 adults aged at least 18 yrs, 53% female	3.3 yrs (0.51)	PA ADJUSTED						Age, sex, BMI, education level, meeting PA guidelines (<30,≥30 min/day at work or in leisure time) smoking status, general health status, cardio-metabolic disease status
				<4	1.00	p=0.001	8529	94	27,915.4	
				4-7	1.12 (0.89-1.42)		20143	302	65,881.9	
				7-<10	1.18 (0.90-1.57)		7843	122	25,510.7	
				>=10	1.65 (1.24-2.21)		5562	122	18,007.8	
				NOT PA ADJUSTED						Age, sex, BMI, education level, smoking status, general health status, cardio-metabolic disease status
				<4	1.00	p<0.001	8531	94	27922.4	
				4-7	1.13 (0.89-1.43)		20147	302	65894.5	
				7-<10	1.21 (0.92-1.60)		7847	122	25522.1	
				>=10	1.75 (1.32-2.32)		5565	122	18017.7	

PA= Physical activity

Data from 595,086 people were included in the meta-analysis. There were 29,162 deaths during 3,565,569 person-years of follow-up. Study sample sizes varied, ranging from 1,906 [30] to 240,819 [11] with follow-up periods ranging from a mean of 2.8 years [10,30] to over 8 years[11,34]. Two studies involved participants aged at least 45 years old[10,34], two involved adults aged at least 50 years[11,30], one study involved adults aged 18 years or more[33], and one study had participants aged 76-81 years old[32]. One study involved women only[32], and over 50% of participants in four of the five other studies were female[10,30,33,34]. One study reported results for men and women separately[34]. All studies ascertained participants’ mortality status through linkage with a regional or national death registry.

Five studies assessed daily total sitting time by self-report[10,11,32-34]. One study used objective measurement of daily sedentary time[30]. All studies assessed daily total sitting time in hours per day grouped into categories. However, the cut-points for the categories were not consistent across the studies (see Table 1). For example, Koster et al [30] divided daily total sedentary time into quartiles, whereas two studies categorized total sitting time as <4, 4-<8, 8-<11, ≥11 h/day[10,32].

All studies adjusted for multiple potential confounding factors including moderate-to-vigorous physical activity. Two studies operationalized physical activity as walking and other moderate-to-vigorous physical activity[10,32], one measured multiple indicators including heavy physical work, walking, exercise and sports[34], one study only assessed moderate-to-vigorous physical activity in leisure time[11], and one study categorized participants as meeting or not meeting physical activity guidelines[33]. Five of the six studies also presented data for models without adjustment for physical activity[10,11,30,32,33], which allowed us to examine associations between daily total sitting time and mortality risk, with and without adjustment for physical activity. 

### Quality assessment

The study appraisal criteria and number of studies scoring a point for each item are presented in Table 2. Study quality scores ranged from 10/15 to 12/15 with a mean percentage agreement of 85.6% on quality ratings between the reviewers. All studies provided information about their objectives, study design, participant sampling and recruitment, measures of total sitting and mortality, data sources and statistical methods. 

**Table 2:** Study quality appraisal criteria and number of studies meeting each criterion *.

**Criterion**		**Inoue 2008 [34]**	**Matthews 2012 [11]**	**van der Ploeg 2012 [10]**	**Koster 2012 [30]**	**Pavey 2012 [32]**	**Chau 2013 [33]**	**n**
1.	Objectives: Are the objectives or hypotheses of the research described in the paper stated?	1	1	1	1	1	1	6/6
2.	Study design: Is the study design presented?	1	1	1	1	1	1	6/6
3a.	Target population: Do the authors describe the target population they wanted to research?	1	0	1	1	1	0	4/6
3b.	Sample: Was a random sample of the target population taken? AND was the response rate 60 percent or more?	0	0	0	0	0	0	0/6
3c.	Sample: Is participant selection described?	1	1	1	1	1	1	6/6
	3d. Sample: Is participant recruitment described, or referred to?	1	1	1	1	1	1	6/6
3e.	Sample: Are the inclusion and/or exclusion criteria stated?	1	1	1	1	0	1	5/6
3f.	Sample: Is the study sample described? (minimum description = sample size, gender, age and an indicator of socio-economic status)	1	1	1	1	1	1	6/6
3g.	Sample: Are the numbers of participants at each stage of the study reported? (Authors should report at least numbers eligible, numbers recruited, numbers with data at baseline and numbers lost to follow up)	1	1	0	0	1	0	3/6
4.	Variables: Are the measures of total sitting and mortality described?	1	1	1	1	1	1	6/6
5a.	Data sources & collection: Do authors describe the source of their data? (e.g., cancer registry, health survey) AND did authors describe how the data were collected? (e.g., by mail)	1	1	1	1	1	1	6/6
5b.	Measurement: Was reliability of the measure(s) of total sitting mentioned or referred to?	0	0	1	0	1	1	3/6
5c.	Measurement: Was the validity of the measure(s) of total sitting mentioned or referred to?	1	1	1	0	1	1	5/6
6a.	Statistical methods: Were appropriate statistical methods used and described, including those for addressing confounders?	1	1	1	1	1	1	6/6
6b.	Statistical methods: Were the numbers/ percentages of participants with missing data for sitting and the health outcome indicated AND If more than 20 percent of data in the primary analyses were missing, were methods used to address missing data?	0	1	0	0	1	0	2/6
	**Score**	12	12	12	10	13	11	

* 0= ‘no’; 1= ‘yes’

### Associations of daily total sitting time with all-cause mortality risk

**Figure 2:**
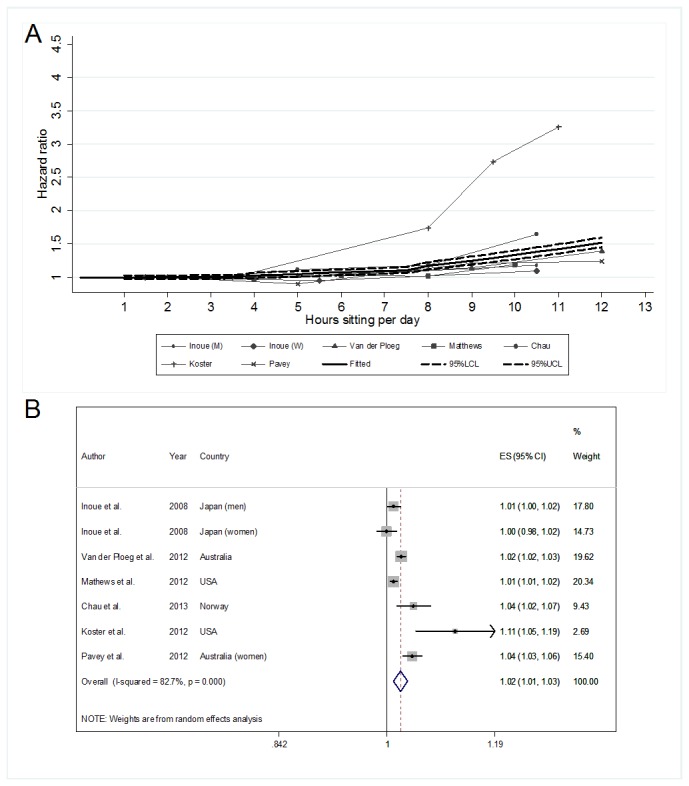
Associations of daily total sitting time with all-cause mortality risk with adjustment for physical activity. **A**: Spaghetti plot of the raw Hazard Ratios (HR) for all-cause mortality from each study sample, by dose of daily total sitting time, with multivariable adjustment including for physical activity. The bold solid line denotes the pooled HR estimate from the fitted spline model and the dotted lines the 95% confidence limits. **B**: Forest plot of the HR per-hour increase in sitting with multivariable adjustment including for physical activity. (n=7 samples from 6 studies).

The analyses involving data with and without adjusting for physical activity involved seven samples from six studies (one study reported results for men and women separately)[34] and five samples from five studies, respectively. The multivariable-adjusted hazard ratios, with adjustment for physical activity, are shown in Figure 2. Figure 2A presents the spaghetti plot of the raw HRs for all-cause mortality from each sample, by dose of daily total sitting time, with the pooled estimate from the fitted spline model, with 95% confidence limits. We observed similar effect sizes for all study samples across dose of sitting, except in the accelerometer study[30], which showed considerably larger hazards of dying from all causes among respondents whose total daily sitting time exceeded the first quartile of the sample (7.6 h/day for men, 7.2 h/day for women). The forest plot of the hazard per-hour increase in sitting, with multivariable adjustment including physical activity, is shown in Figure 2B. For each study the HRs, with 95% CIs, are shown, along with the percent weighting. We found a pooled hazard ratio of 1.02 (95%CI: 1.01-1.03). Heterogeneity was high (I^2^=82.7%) and statistically significant (p<0.001). A sensitivity analysis excluding the results of the Koster et al [30] study did not change the pooled estimate (HR=1.02; 95% CI: 1.01-1.03), nor improve heterogeneity (I^2^=81.0%, p <0.001).

**Figure 3:**
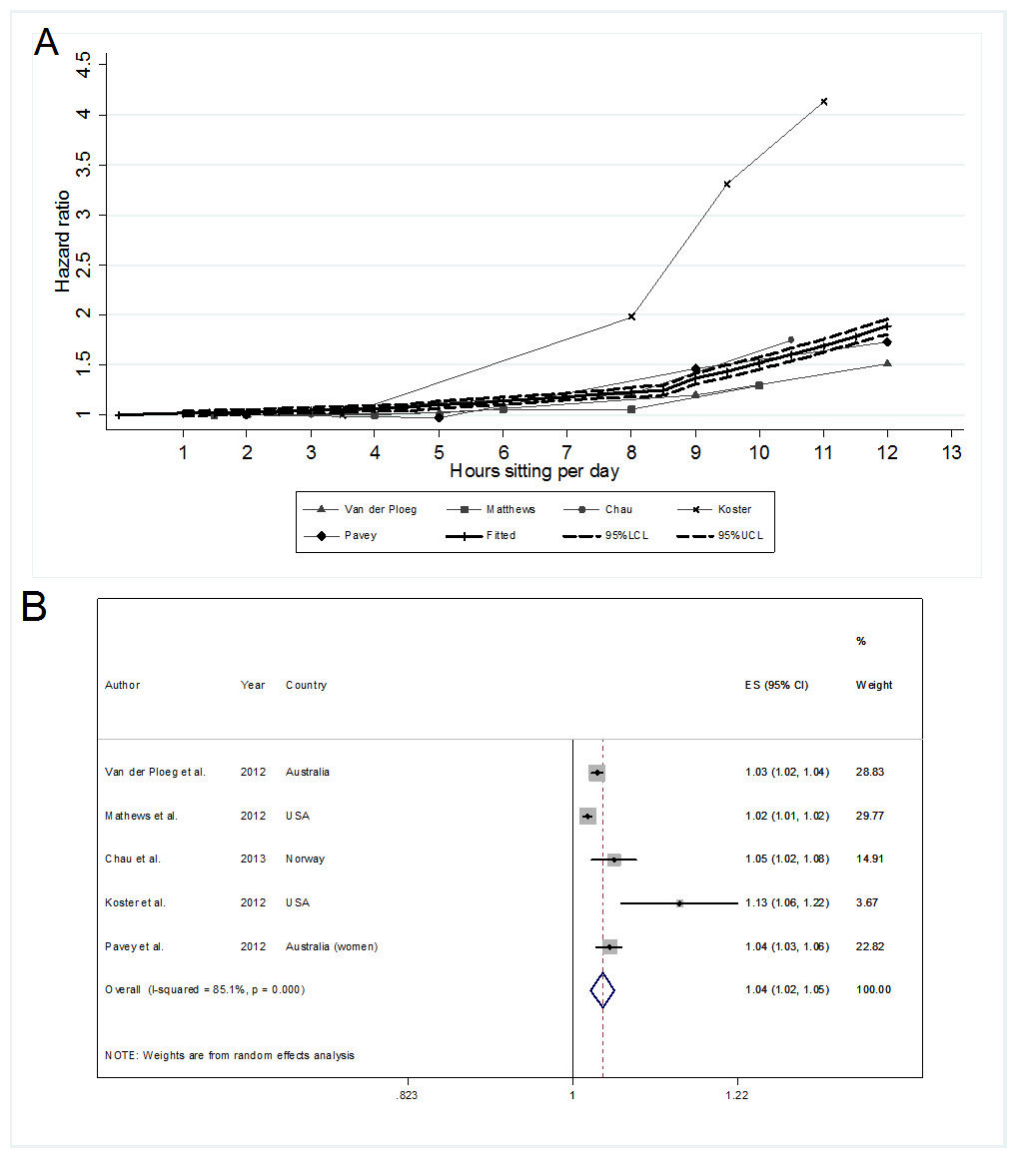
Associations of daily total sitting time with all-cause mortality risk with no adjustment for physical activity. **A**: Spaghetti plot of the raw Hazard Ratios (HR) for all-cause mortality from each study sample, by dose of daily total sitting time, with multivariable adjustment but not for physical activity. The bold solid line denotes the pooled HR estimate from the fitted spline model and the dotted lines the 95% confidence limits. **B**: Forest plot of the HR per-hour increase in sitting with multivariable adjustment but not for physical activity. (n=5 samples from 5 studies).

Figure 3 presents the results for the analyses involving data with no correction for physical activity. We observed similar patterns to those seen for data with adjustment for physical activity (Figure 2). The pooled HR of 1.04 (95% CI: 1.02-1.05) was higher than that for the physical activity adjusted analyses, and heterogeneity was also high (I^2^=85.1%, p<0.001). 

### Evidence of dose-response

Initial linear random effects analyses suggested that the association between daily total sitting time and all-cause mortality was not linear in models with adjustment for physical activity (HR=1.03; 95% CI: 0.98-1.08) and without physical activity adjustment (HR=1.05; 95% CI:0.98-1.13). The goodness-of-fit test suggested that the linear dose-response models did not fit the data well with adjustment for physical activity (χ^2^_(19)_ = 71.02, p<0.001) and without physical activity adjustment (χ^2^_(15)_ = 91.37, p<0.001). 

Multiple non-linear spline models were subsequently fitted and are shown graphically by the bold solid lines in Figures 2A and 3A. Estimates at specific doses take into account the trends at preceding “knots”, and it is best to read estimates from the figures provided. For the physical activity adjusted data, the best fitted spline model was with knots at >3 and >7 h/day (χ^2^_(17)_ =22.67, p=0.160) (Figure 2A). For every 1-hour increment of sitting in intervals 0-3, >3-7 and >7 h/day total sitting time, the HRs were 1.00 (95% CI: 0.98-1.03), 1.02 (95% CI: 0.99-1.05) and 1.05 (95% CI: 1.02-1.08) respectively. For data without adjustment for physical activity level, the trend estimates were similar but with slightly steeper slopes. The best fitted spline model was with knots at >4 and >8 h/day (χ^2^_(13)_=17.85, p=0.163) (Figure 3A). For every 1-hour increment of sitting in intervals 0-4, >4-8 and >8h/day total sitting time, the HRs were 1.02 (95% CI: 0.99-1.04), 1.02 (95% CI: 1.00-1.04), and 1.08 (95% CI: 1.05-1.11), respectively. Spline modeling with knots at >3 and >7 h/day did not fit the data without adjustment for physical activity well (χ^2^_(13)_ =24.27, p=0.029). For example, based on the dose-response spline models (Figures 2A and 3A), an adult sitting for 10 h/day would have 34% (HR=1.34, 95% CI: 1.28-1.40) and 52% (HR=1.52, 95% CI: 1.46-1.58) increased all-cause mortality risk with and without adjusting for physical activity, respectively.

The overall weighted PAF for all-cause mortality attributable to total daily sitting time, after taking physical activity into account, was estimated to be 5.9%. Individual study PAFs ranged from 1.9% for the largest study with self-report data [11] to 58% for the smaller objective measure study by Koster and colleagues[30]. When the Koster et al study was excluded, the overall weighted PAF for all-cause mortality attributable to total daily sitting time was 4.4% (range: 1.9% - 15.6%), after accounting for physical activity. 

### Evidence of publication bias

The small number of studies and presence of significant heterogeneity precluded formal testing for publication bias[35]. However, visual inspection of the forest and funnel plots (Figure S1) suggest that there may be some publication bias present, with larger cohort studies reporting the smaller effects[36].

## Discussion

This meta-analysis of data from 595,086 participants in six prospective studies on the associations between total sitting and all-cause mortality found that each additional hour of daily sitting is associated with an overall 2% increased risk of all-cause mortality after physical activity is taken into account. However, the association between sitting time and all-cause mortality risk was found to be non-linear, with a 5% increased risk of all-cause mortality for each 1-hour increment in sitting time per day for adults sitting >7 h/day, after accounting for multiple covariates including physical activity. However, there was no evidence of higher mortality risk per 1-hour increment of sitting at daily total sitting times 0-3 and >3-7 h/day. When physical activity was not taken into account, total sitting >4-8 and >8 h/day were both associated with significantly higher risk of dying (2% and 8% per 1-hour increment in sitting time per day, respectively). This suggests that physical activity partly attenuates the deleterious associations between total sitting time and all-cause mortality, especially in those in the highest sitting time category.

Our findings suggest that the overall multivariable-adjusted effect of daily total sitting time on all-cause mortality risk is relatively small. Nonetheless, all-cause mortality risk appears to increase progressively as people sit more during the day, with physical activity having a partially protective effect, especially when sitting time is high. Our dose-response modeling estimated the risk of all-cause mortality for sitting 10 h/day to be 34% and 52% higher than sitting for 1 h/day when physical activity was and was not taken into account, respectively (Figures 2A and 3A).

The findings of this meta-analysis are consistent with a meta-analysis of three studies on TV-viewing and mortality[13], which showed a pooled HR for all cause mortality of 1.13 per two hour increase of TV-viewing. Other meta-analyses have shown pooled HRs of 1.18-1.45 [15] and 1.49 [18] for the group with the highest level of sitting or sedentary time compared with the lowest group, respectively. However, both studies combined different measures of sedentary behavior (TV-viewing mostly, and sitting time) in their analyses, and neither study examined dose-response relationships for daily total sitting time. Furthermore, earlier meta-analyses did not compare associations between sitting and mortality, with and without adjusting for physical activity. Our meta-analysis demonstrates the attenuation of mortality risk for daily sitting time after taking physical activity into account, by presenting results for models with and without adjustment for physical activity.

Five of the six studies included in this meta-analysis assessed total sitting time through single-item self-report measures, and measurement error in reported sitting time could have led to misclassification and attenuation of the risk estimates observed in these studies[31,37]. This may have resulted in an underestimation of the strength of the underlying association. The only study that used an objective measure of sedentary behavior showed a much stronger association with all-cause mortality[30]. However, this was also the smallest study (145 deaths) and the average follow up was relatively short (2.8 years), which increases the risk of reverse causality. A relatively short follow up period was also a limitation of two other studies[10,33]. Nonetheless, all three studies that had a short follow up period conducted sensitivity analyses in order to check for potential reverse causality (at least by excluding people who died in the first year), and all three reported similar associations between sitting time and all-cause mortality in their sensitivity analyses[10,30,33].

Although our pooled estimates were based on prospective cohort studies, with analyses that corrected for multiple potential confounders, there remains the possibility of unmeasured confounding or reverse causality in the included studies. All studies in this meta-analysis adjusted their analyses for age and physical activity, while four out of six studies also adjusted for sex, and five studies for education and/or smoking (Table 1). Other confounding variables that were taken into account in the studies in this meta-analysis included race, alcohol intake, geographic location, BMI, occupation and marital status. Further, four studies adjusted for self-rated health or existing health conditions and physical limitations, which is an important consideration in the complex relationship between sitting and health, as sick people are likely to sit more and have higher risk of dying prematurely. One study stratified analyses by sex, age group, BMI categories and pre-existing chronic illness, and observed results consistent with those found for the whole sample[10]. Another study conducted sensitivity analyses by excluding participants with mobility limitations, cardio-metabolic disease, and cancer and found that the associations between sedentary time and mortality were maintained[30]. Two studies also excluded participants with poor self-rated health at baseline or pre-existing chronic illness from their analyses[11,32]. The range of adjustments and exclusions in the studies in this meta-analysis may partly account for the significant heterogeneity observed, although the potential for reverse causality cannot be excluded.

The major strength of this study is that it is the first meta-analysis of dose-response relationships between total daily sitting time and mortality risk with and without adjustment for physical activity. Other strengths are the inclusion of data from population based prospective cohort studies, with relatively consistent measurement of daily sitting time in hours per day. 

The main limitation of this meta-analysis is the small number of studies included, although this was greater than in some previous meta-analyses[13,15]. This highlights the emerging interest in this field, and the need for more longitudinal studies to include more comprehensive and better measures of sedentary behavior in future studies[38]. It will, however, take some time for new studies to accumulate sufficient exposure time to be able to add to the results presented here. 

In light of the increasing population prevalence of sedentary behavior[39], our findings have implications for public health. A recent multinational study has shown that 25% of people in developed and developing countries sit for 8 or more hours per day[7]. We estimated that 5.9% of deaths could be attributed to daily total sitting time, even with physical activity taken into account. This estimate is similar to that reported by the World Health Organization for other major risk factors such as tobacco use (8.7%), physical inactivity (5.5%) and overweight and obesity (4.8%)[40], and suggests that if daily sitting time were reduced the beneficial effect on population health could be comparable to that achieved for reducing smoking, inactivity or overweight and obesity.

Our findings suggest that it is timely to develop public health guidelines for sitting, similar to those for other behavioral risk factors. Although current physical activity guidelines, such as those from the USA [41] and UK [42], include advice for adults to reduce the amount of time spent sedentary or sitting for prolonged periods, they do not indicate how much sitting is harmful for health. The results of this meta-analysis provide a starting point for making more specific recommendations about how much sitting is associated with adverse health outcomes and the potential health benefits of reducing daily total sitting time, in addition to those accrued through engaging in moderate-to-vigorous physical activity. Future studies should now examine associations between sitting time and disease-specific morbidity and mortality.

In conclusion, this meta-analysis of data from prospective cohort studies suggests that higher amounts of daily total sitting time are associated with greater risk of dying from all-causes. Overall, each hour of daily sitting time was associated with a 2% increase in all-cause mortality risk, after taking the protective effects of physical activity into account. The risk appears to increase significantly when adults sit for more than 7 h/day; by 5% for each 1-hour increment in daily sitting time, when the effects of physical activity are taken into account. The findings from this study provide a starting point for identifying a threshold on which to base sedentary behavior recommendations for overall sitting time. Until then, public health and clinical recommendations should continue to advise adults to sit less throughout the day, in addition to physical activity guidelines.

## Supporting Information

Checklist S1 **PRISMA checklist.**
(DOC)

Figure S1 **Funnel plots for prospective cohort studies of daily total sitting time and all-cause mortality risk.** **A**: Studies with multivariable adjustment including for physical activity (n=7 samples from 6 studies). **B**: Studies with multivariable adjustment but not for physical activity (n=5 samples from 5 studies).
(TIF)
