# Supplementary figures and images for: Daily Sitting Time and All-Cause Mortality: A Meta-Analysis
Source: PLoS One. 2013 Nov 13;8(11):e80000. doi: 10.1371/journal.pone.0080000 (PMC3827429; doi:10.1371/journal.pone.0080000)

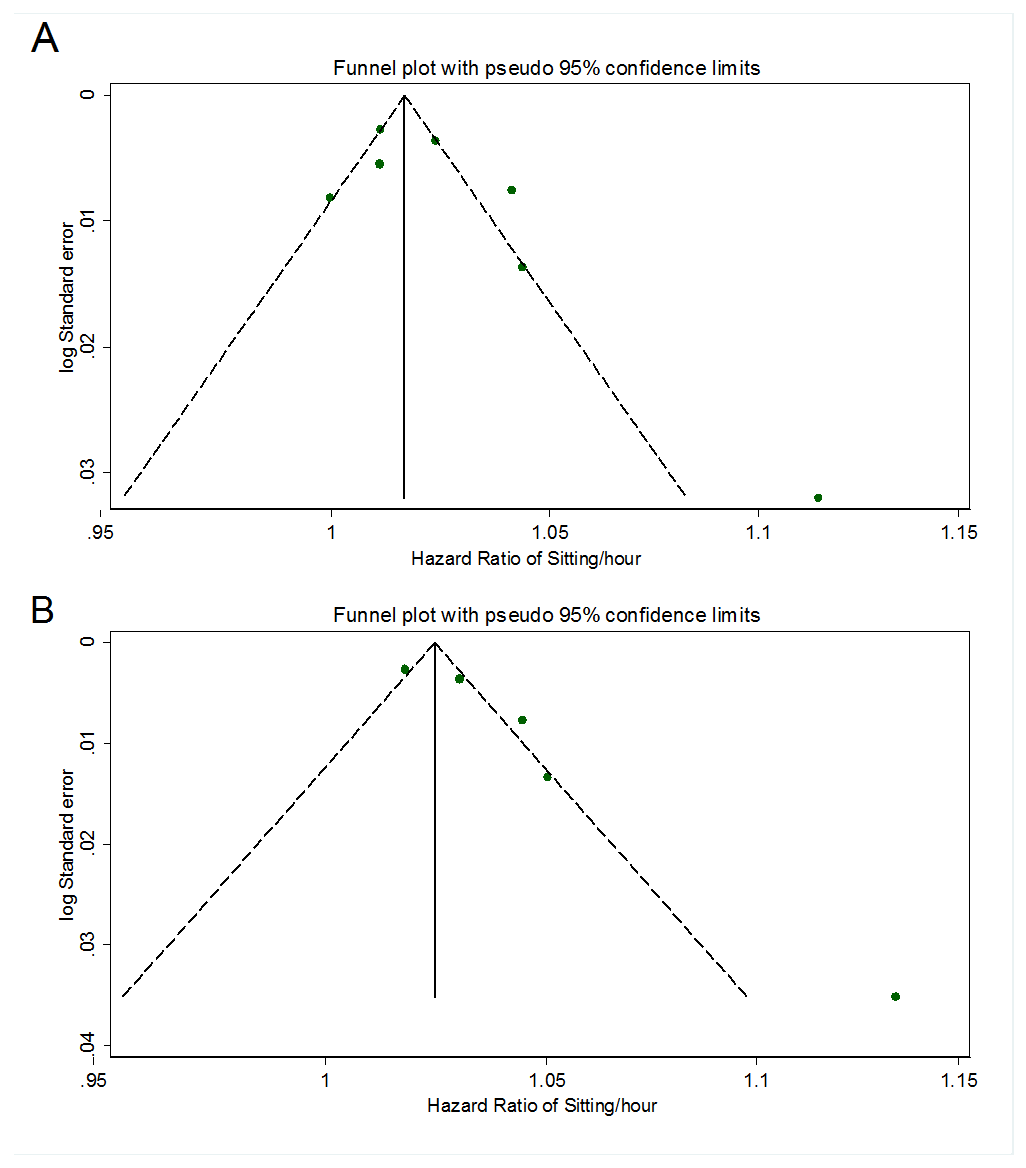

Supplement: Figure S1 — Funnel plots for prospective cohort studies of daily total sitting time and all-cause mortality risk. A: Studies with multivariable adjustment including for physical activity (n=7 samples from 6 studies). B: Studies with multivariable adjustment but not for physical activity (n=5 samples from 5 studies). (TIF) [file pone.0080000.s002.tif]
